# Current Challenges for HER2 Testing in Diagnostic Pathology: State of the Art and Controversial Issues

**DOI:** 10.3389/fonc.2013.00129

**Published:** 2013-05-21

**Authors:** Anna Sapino, Margherita Goia, Daniele Recupero, Caterina Marchiò

**Affiliations:** ^1^Department of Medical Sciences, University of TurinTurin, Italy

**Keywords:** HER2, truncated HER2, CEP17 amplification, HER2 mutations, diagnosis, therapy, test

## Abstract

HER2 overexpression and anti-HER2 agents represent probably the best story of success of individualized therapy in breast cancer. Due to the important therapeutic implications, the issue under the spotlight has been, since ever, the correct identification of true HER2 positivity on tissue specimens. Eligibility to anti-HER2 agents is strictly dependent on the demonstration of HER2 overexpression (by immunohistochemistry) or of *HER2* gene amplification by *in situ* techniques (fluorescence *in situ* hybridization, FISH), however there are controversial issues involving cases with “equivocal” HER2 status based on conventional techniques (about 20% of specimens). In terms of HER2 expression a major debate is the presence of full-length and truncated forms of the protein and controversial clinical data have been reported on the therapeutic implications of these HER2 fragments. In terms of *HER2* gene assessment, the occurrence of amplification of the chromosome 17 centromeric region (CEP17) has been proven responsible for misleading *HER2* FISH results, precluding anti-HER2 based therapy to some patients. Finally HER2 activating mutations have been recently described as a biological mechanisms alternative to *HER2* gene amplification. In this review we will focus on the controversies that pathologists and oncologists routinely face in the attempt to design the most tailored treatment for breast cancer patients. We will focus on the *HER2* gene and on the protein, both at technical and interpretational levels.

## Introduction

HER2, a member of the human epidermal growth factor receptor family, is an orphan tyrosine kinase receptor that is overexpressed in 15–20% of breast cancers and these carcinomas show a poor prognosis (Slamon et al., 1987; Marchiò and Reis-Filho, 2008). HER2 overexpression is a direct result of gene amplification in ∼95% of cases and represents perhaps the best target for individualized therapy because it has been shown to be a tumor driver and an excellent example of “oncogene addiction” (Slamon et al., 1987; Marchiò and Reis-Filho, 2008). The humanized mouse monoclonal antibody Herceptin^®^ (namely trastuzumab) targeting the extracellular domain (ECD) of HER2 is nowadays offered to breast cancer patients in advanced, adjuvant, and neoadjuvant settings in association with chemotherapy. Moreover, in the case of metastatic HER2+ breast cancer a tyrosine kinase inhibitor, lapatinib (Geyer et al., 2006), as well an antibody targeting HER2-HER3 dimerization, pertuzumab (Baselga et al., 2012), have also been approved by U.S. Food and Drug Administration (FDA) for treatment in combination with chemotherapy.

The introduction of such a tailored therapeutic option has had a tremendous impact on the natural history of HER2-positive disease over the years and, due to the important therapeutic implications, the issue under the spotlight has been, since ever, the correct identification of true HER2 positivity on tissue specimens. The relevance of such assessment on HER2+ breast cancer care will be further enhanced if trastuzumab is administered to patients in monotherapy. Indeed, studies to improve drug effectiveness of trastuzumab led to the development of trastuzumab emtansine (i.e., trastuzumab-DM1, T-DM1), an antibody-drug conjugate consisting of trastuzumab linked to the cytotoxin mertansine (DM1). T-DM1 is designed to target and inhibit HER2 signaling and to deliver the chemotherapy directly inside HER2+ cancer cells. At present T-DM1 is currently being employed in clinical trials (Bose et al., 2013; Peddi and Hurvitz, 2013): a phase II randomized trial of T-DM1 in the front-line metastatic breast cancer setting has revealed promising activity and improved safety compared with standard chemotherapy plus trastuzumab (Hurvitz et al., 2012); in addition a phase III trial in patients with trastuzumab-pretreated metastatic breast cancer showed T-DM1 to be associated with prolonged progression-free and overall survival compared with lapatinib plus capecitabine (Verma et al., 2012).

T-DM1 represents a paradigm shift in the treatment of breast cancer patients and in such a scenario it is mandatory to precisely recognize HER2+ carcinomas, because patients will not be administered any other chemotherapeutic agents and response to treatment will strictly rely on pathological assessment of HER2.

## HER2 Study in Diagnostic Practice Today: A Pragmatic Overview

Based on the premises above, it is not surprising that in a standard day of routine diagnostic practice HER2 scoring, along with the assessment of other prognostic and predictive factors, is undoubtedly one of the topic moments in terms of breast cancer pathology. Before getting down into the nitty gritty details of the methodology there are some questions that need to be answered regarding where and when we should test HER2. As detailed above, anti-HER2 agents are currently being offered as a standard of care both in early-stage and metastatic breast cancer. In terms of neoadjuvant setting one caveat should be spelled out in terms of adequacy of specimens. Neoadjuvant therapy is planned for patients with locally advanced carcinomas or with tumors primarily not suitable for breast-conserving surgery, thus the first aim of this therapeutic option is to downsize the disease burden and possibly to achieve pathologic complete response (pCR) (Marchio and Sapino, 2011). In order to target the disease it is mandatory to have a proper sampling of the lesion to account for tissue heterogeneity at histological and immunophenotypical level, which represents a crucial factor for treatment decision making and finally for the success of the therapy. Although international guidelines are not available on the topic, in our routine experience sampling of different tumor areas by core biopsy (three biopsies on average) is helpful to define the precise nature of the lesion, the different tumor histological types present in the mass, and the heterogeneity in the expression of predictive markers (Marchio and Sapino, 2011). Following neoadjuvant treatment surgery is performed and proper histological documentation of residual disease is mandatory in association with re-testing of prognostic and predictive factors (Marchio and Sapino, 2011).

Other hot topic about timing of HER2 testing is represented by metastatic disease. Several studies have demonstrated a discrepancy between primary and metastatic tumors (Gancberg et al., 2002; Regitnig et al., 2004; Gong et al., 2005; Fabi et al., 2011). Based on the fact that change in the phenotype may mean the possibility to add a precious and potentially life-saving therapeutic option, HER2 reassessment in metastatic lesions should be carefully taken into account, whenever feasible, especially for metastases coming from primary hormone receptor-positive breast cancer (Fabi et al., 2011).

### Best laboratory practice

The methodological approach depends on the laboratory organization, as two FDA-approved techniques are available. Although they can be used indiscriminately, usually the first step is represented by the assessment of HER2 protein overexpression in immunohistochemistry (IHC), which contemplates a well known four-tier scoring system. Scores 0 and 1+ are considered as negative (no eligibility to anti-HER2 treatment), score 3+ is considered as positive (eligibility to anti-HER2 treatment), whereas score 2+ [up to 24% of all cases (Lee et al., 2011)] constitutes a gray zone in which further tests are needed (Wolff et al., 2007). However, the IHC scoring system is not so robust and the cut-off value (percentage of cells to be positive) across different score classes has been changing over time. Recently Perez et al. (2012) call for the attention on the differences between the FDA scoring system for HER2 expression (10% cut off) used in some adjuvant trastuzumab trials and the one proposed by the ASCO/CAP guidelines (30% cut off), showing that the latter determined a certain rate of false negative HER2 tumors. On the other hand, the answer of some of the ASCO/CAP guideline authors (Wolff et al., 2012) was that this scoring system reduces the number of false-positive results. In any case the moderate expression of HER2 (2+) even without *HER2* gene amplification is a negative prognostic factor in early breast cancer (Rossi et al., 2012).

Regarding *HER2* gene status, three FDA approved *in situ* hybridization techniques are available: fluorescence *in situ* hybridization (FISH), chromogenic *in situ* hybridization (CISH), silver *in situ* hybridization (SISH). Very recently a “fast FISH” has been developed (IQFISH) (Matthiesen and Hansen, 2012): this technology exploits alternative solvents and a new hybridization buffer that reduces the required hybridization time to 1 h, thus shortening the turnaround time from sample to diagnosis without affecting output results (a concordance of 98% with conventional FISH has been proven) (Matthiesen and Hansen, 2012).

For *in situ* hybridization analysis two scoring systems with distinct thresholds for *HER2* gene copy number and *HER2*/CEP17 ratio are available. Indeed, the FDA and ASCO/CAP schemes for HER2 evaluation differently select patients for trastuzumab therapy. Amplification is defined as: (i) *HER2*/CEP17 >2 or *HER2* copy number >4 according to FDA (Jacobs et al., 1999; Birner et al., 2001; Brunelli et al., 2008); (ii) *HER2*/CEP17 >2.2 or *HER2* copy number >6 according to ASCO/CAP. The latter scoring system has introduced the “equivocal” range, in which fall those cases harboring a *HER2* copy number between 4 and 6 or a *HER2*/CEP17 between 1.8 and 2.2. Of note, the minimal thresholds of >4 gene copy number is required as replicating cells (G2 phase, in anticipation of cell division) will have four copies of chromosome 17 and *HER2* gene, therefore breast tumors with normal *HER2* status but high proliferative activity may have a mean *HER2* copy number up to 4 (Ross et al., 2003; Szollosi et al., 2005).

From a technical standpoint, in order to guarantee the best IHC and FISH performance, technicians as well as molecular biologists, and pathologists are demanded to work in close collaboration and key points in pre-analytical, analytical, and post-analytical phases of HER2 testing have been identified, as largely detailed in the ASCO/CAP guidelines (Wolff et al., 2007). Issues about reproducibility and reliability of HER2 testing have always been a matter of debate among pathologists (Wolff et al., 2007) and some of the major problems affecting such reproducibility are discussed here below.

As an example, for the pre-analytical phase bold claims have been recently made about the impact cold ischemia time (i.e., time to fixation) may have on HER2 testing (Pekmezci et al., 2012; Yildiz-Aktas et al., 2012a,b). The shorter the cold ischemia time the better is the quality of HER2 staining (Pekmezci et al., 2012; Yildiz-Aktas et al., 2012a,b), and results are poorer for non-refrigerated samples (Yildiz-Aktas et al., 2012a). Ideally cold ischemic time should not exceed 1 h, then, upon sampling formalin fixation (10% neutral buffered formalin) should be applied within a time frame comprised between 6 and 48 h (Wolff et al., 2007). However, controlling the time of fixation is a difficult matter, because immersion in formalin of a large surgical specimen does not mean initiation of fixation of a tumor. Our group has successfully explored the under vacuum sealing of large specimens and cooling at 4°C for transport from the surgical theater to the pathology lab as a method that allows monitoring exactly the time of ischemia and of initiation of fixation and guarantees an optimal preservation of antigens (Bussolati et al., 2011; Comanescu et al., 2012).

In terms of analytical phase the availability of distinct antibodies and their specificity can take part in affecting reproducibility of results. FDA-approved anti-HER2 antibodies for IHC (Wolff et al., 2007) are directed against the intracellular domain. In routine diagnosis it is suggested to use kit preparations such as: pathway HER2 (clone 4B5; Ventana Medical Systems Inc., Tucson, AZ, USA), HercepTest (Dako, Glostrup, Denmark), and Oracle HER2 (clone CB11; Leica Microsystems GmbH, Wetzlar, Germany). In a recent work it has been shown that all three antibodies react with HER2 proteins and peptides in IHC stainings, ELISA, and immunoblotting. However, while HercepTest shows no cross-reactivity with other proteins of the HER family, the others cross-react with HER4 (Schrohl et al., 2011). Antibodies targeting the ECD are commercially available (such as Tab250, Invitrogen, San Diego, CA, USA), however none of them recognizes the trastuzumab binding site (epitope in the cysteine rich region of the IV domain, in proximity of the juxtamembrane region). The only antibody targeting the trastuzumab binding site is the 4D5 (Genentech, Inc., San Francisco, CA, USA), the murine monoclonal, later humanized as trastuzumab (Herceptin^®^), which is not commercially available. In our lab we developed a biotinylated form of Herceptin^®^, the BiotHER (Bussolati et al., 2005; Sapino et al., 2007), which can be used in formalin fixed paraffin embedded (FFPE) tissues. BiotHER positivity, in a large series of advanced breast cancers treated with Herceptin^®^, showed a significant correlation with response (Bussolati et al., 2005; Sapino et al., 2007).

### Focus on post-analytical phase: Intra-tumoral heterogeneity

Interpretation and reporting are crucial moments, especially when dealing with *in situ* hybridization. A real challenge and widely discussed matter of debate is represented by intra-tumoral HER2 heterogeneity. If on one side decisions for therapy require a yes/no answer, in the other HER2 status derives from a continuum of gene copy number and protein expression (Oakman et al., 2010), especially in equivocal cases. As said above, the widely adopted ASCO/CAP guidelines (Wolff et al., 2007) have implemented stricter thresholds (30% *versus* 10%) to improve detection reliability, accuracy, and reproducibility of IHC and FISH with the final aim to ameliorate the concordance between IHC and FISH analyses, leading to a narrower selection of population eligible for trastuzumab treatment (Wolff et al., 2007). In addition, they do not take a position about intra-tumoral heterogeneity, as they read “*If genomic heterogeneity of HER2 gene amplification is found, it must be specifically reported* (Hicks and Tubbs, 2005; Wolff et al., 2007)*. No consensus recommendations exist at this time for handling of genomic heterogeneity*” (Wolff et al., 2007). HER2 genetic heterogeneity is defined as the presence of more than 5% but less than 50% of infiltrating tumor cells with a *HER2*/CEP17 ratio higher than 2.2 (Vance et al., 2009).

According to this definition (Vance et al., 2009), HER2 heterogeneity ranges between 5% (Vance et al., 2009) and 15% (Ohlschlegel et al., 2011) of total cases tested and seems to be most frequent (up to 27%) in breast carcinomas with an equivocal (2+) HER2 score (Ohlschlegel et al., 2011). In a recent study, genetic heterogeneity was associated with a negative *HER2* amplification status in 16% of all carcinomas and 42% of HER2 (2+) carcinomas, respectively (Ohlschlegel et al., 2011). This means that the group of HER2 amplification-negative carcinomas comprise a subgroup of HER2 genetic heterogeneity-positive carcinomas that harbor a significant subpopulation (>5%) of tumor cells with *HER2* amplification but do not qualify for trastuzumab treatment based on current recommendations (*HER2*/CEP17 ratio – calculated on the overall population – below 2.2) (Ohlschlegel et al., 2011).

About heterogeneity a distinction should be made, as in general two main types of such a phenomenon can be encountered and may have distinct implications. The first one is represented by presence of two distinct populations of cells (i.e., two different clones of cancer cells within a lesion), one completely negative for HER2 and the other clearly positive (*HER2* amplified) (Oakman et al., 2010). Such a scenario may be assimilated to the so-called “focal HER2 amplified clones” (FHACs), which have been reported in an N9831 substudy (Miller et al., 2004). FHACs were defined as having 2–40% of cells with unequivocal amplification (cells with >10 *HER2* signals or *HER2*/CEP17 ratio >5, regardless of overall *HER2*/CEP17 ratio) (Miller et al., 2004) and were detected particularly in tumors with discordance between HER2 status by IHC and FISH (21% of IHC 0–1/FISH-amplified and 30% of IHC2+/FISH-amplified cases contained FHACs) (Miller et al., 2004). The therapeutic implications of this finding have been explored in 91 patients from N9831 with FHAC and compared with 1571 patients with diffuse HER2 amplification (Sukov et al., 2009) and a similar trastuzumab benefit was seen for patients with HER2 amplification, either diffuse or focal (Oakman et al., 2010).

The second type of heterogeneity, which leads to considerable troubles in FISH reporting, is represented by those tumors in which scattered *HER2* amplified cells are identified within a homogeneous background of cells that substantially lack HER2 gain or amplification (Oakman et al., 2010). The biological meaning of such a scenario is much more controversial and probably recommendations would benefit from definition of a cut-off for the cell population harboring *HER2* amplification.

## “*News and Views*” from Experimental Studies on HER2

In this paragraph we will focus on recent experimental studies that have brought to the forefront phenomena that can affect HER2 identification, interpretation/reporting, and response to treatment. In terms of HER2 expression a major debate is represented by the presence of full-length or truncated/fragmented forms of the protein. In terms of *HER2* gene assessment, the recent demonstration of occurrence of amplification of the centromeric region of chromosome 17 (CEP17) can be responsible for misleading *HER2* FISH results, precluding a potentially life-saving treatment to breast cancer patients.

### Truncated HER2 protein

Although the *HER2* gene encodes for the full-length membrane-spanning receptor p185^HER2^, approximately 30% of HER2+ tumors express a variety of receptor fragments sized between 90 and 115 kDa, collectively known as p95^HER2^ carboxy-terminal fragments (CTFs) (Parra-Palau et al., 2010; Recupero et al., 2013). p95^HER2^ is described to be consistently found in a subset of HER2-positive carcinomas (Arribas et al., 2011; Zagozdzon et al., 2011), i.e., in the presence of high levels of p185^HER2^ (Recupero et al., 2013) and therefore in *HER2* amplified cases (Recupero et al., 2013).

Two main mechanisms can lead to the formation of p95^HER2^ fragments: the proteolytic cleavage mediated by alpha-proteases (Codony-Servat et al., 1999) and translation of the mRNA encoding HER2 from internal initiation codons (Christianson et al., 1998; Anido et al., 2006; Arribas et al., 2011). Proteolytic cleavage results in the formation of two receptor fragments, i.e., the soluble p105 fragment of ECD (released in the extracellular compartment) and the oncogenic 95- to 100-kDa p95^HER2^ fragment (648-CTF), which is anchored to the plasma membrane. A disintegrin and metalloproteinase 10 (ADAM10) has been identified as a major source of shedding of the ECD of HER2 in HER2+ breast cancer cells (Liu et al., 2006). By alternative initiation of translation two p95^HER2^ fragments are generated, of 100- to 115-kDa (611-CTF) and 90- to 95-kDa (678-CTF), respectively. Pedersen et al. (2009) have analyzed the activity of the individual p95^HER2^ fragments and showed that the soluble intracellular 90-to 95-kDa fragment (678-CTF) was inactive, despite having an intact kinase domain (Pedersen et al., 2009), whereas the membrane-bound p95HER2 fragments were active. Although the activity of the 95- to 100-kDa fragment (648-CTF) was comparable with that of the full-length receptor, expression of the 100- to 115-kDa fragment (611-CTF) led to a much more rapid and acute activation of different signaling cascades (Pedersen et al., 2009). As a result, expression of the 100- to 115-kDa p95^HER2^ fragment (611-CTF) leads to the regulation of a specific set of genes not regulated by full-length HER2 (Pedersen et al., 2009) that are involved in the metastatic progression (Pedersen et al., 2009). In addition, this fragment has been recently shown to play a role in the intertalk with the estrogen receptor (ER) during malignant progression: it has been demonstrated that 611-CTF induces resistance to anti-estrogen therapy and a more pronounced down-modulation of ER than that induced by full-length HER2 (Parra-Palau et al., 2010; Recupero et al., 2013).

**Figure 1:**
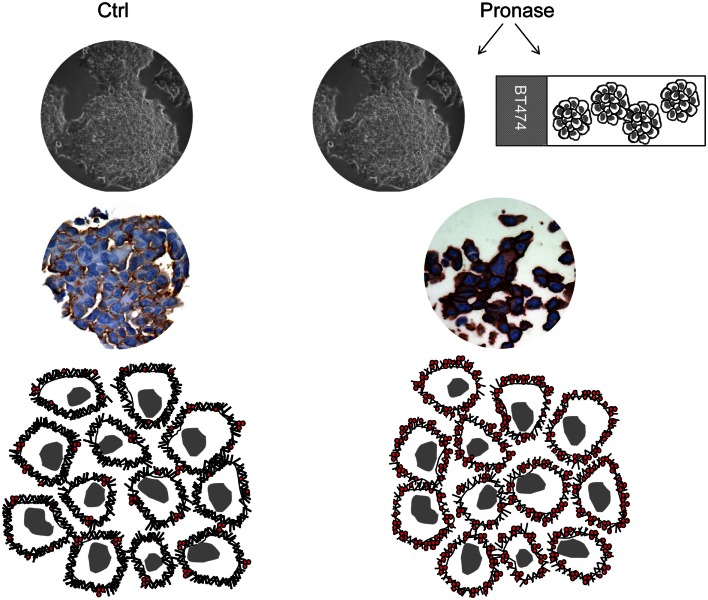
**Steric hindrance phenomenon**. Immunohistochemistry for the trastuzumab binding site (BiotHER staining) shows higher intensity in cell block sections of BT474 cells treated with 0.1% pronase and in pronase-treated (as “antigen retrieval”) cell block sections of BT474 cells. This is likely to be due to a matter of steric hindrance, according to which if antigen molecules are closely “packed” on the cell surface, spatial interference results in a greater likelihood of reduced antibody binding.

Overall, p95^HER2^ is clinically associated with aggressive disease, poor prognosis, and, by lacking the trastuzumab binding epitope, it has been implicated also as a mechanism of resistance to the antibody (Molina et al., 2001, 2002; Saez et al., 2006; Scaltriti et al., 2007; Guarneri et al., 2012). The latter observation has been recently called into question by the results from the neoadjuvant GeparQuattro study (chemotherapy plus trastuzumab treatment), which has showed that p95^HER2^ expression, measured by using a monoclonal antibody that specifically recognizes the 611-CTF in IHC, indicates response to the neoadjuvant trastuzumab-based regimen (Loibl et al., 2011). The results from the GeparQuattro study open a new, yet controversial, perspective in terms of trastuzumab-based therapy, that may be explained, at least in part, by recently reported experimental data (Recupero et al., 2013). Indeed, we have analyzed a series of breast carcinoma for p95^HER2^ by using western blot and compared the results with IHC for both intracellular domain (CB11) and trastuzumab binding site [by using BiotHER (Bussolati et al., 2005; Sapino et al., 2007)]. Surprisingly we observed a significantly higher percentage of 3+ scored cells (with both antibodies) in p95^HER2^-positive cases, suggesting that p95^HER2^ does not compromise the immunohistochemical detection of HER2 and does not affect the trastuzumab binding site (Recupero et al., 2013). A possible explanation for this unexpected increase in immunoreactivity of the anti-HER2 antibodies in the presence of p95^HER2^ may be the reduction of the antigen “steric hindrance” (Kent et al., 1978; Recupero et al., 2013): if the antigen molecules are closely “packed” on the cell surface, spatial interference may result leading to a greater likelihood of reduced antibody binding. This hypothesis was proven by experimental studies in an *in vitro* model of p95^HER2^ expressing breast cancer cells obtained via culturing the HER2+ BT474 cells with pronase, a cocktail of 10 proteases. Indeed, short-term pronase digestion of BT474 cells (i) produced two HER2 fragments (of 95 and 150 kDa), (ii) increased the binding affinity of trastuzumab, (iii) reduced the rate of HER2-HER3 dimers, (iv) and did not interfere with pertuzumab-binding capacity (Recupero et al., 2013). We may therefore conclude that p95^HER2^ is likely to foster a reduction of the antigen “steric hindrance,” thus facilitating the binding capacity of trastuzumab (Figure 1).

### Facts and artifacts about chromosome 17 polysomy

**Figure 2:**
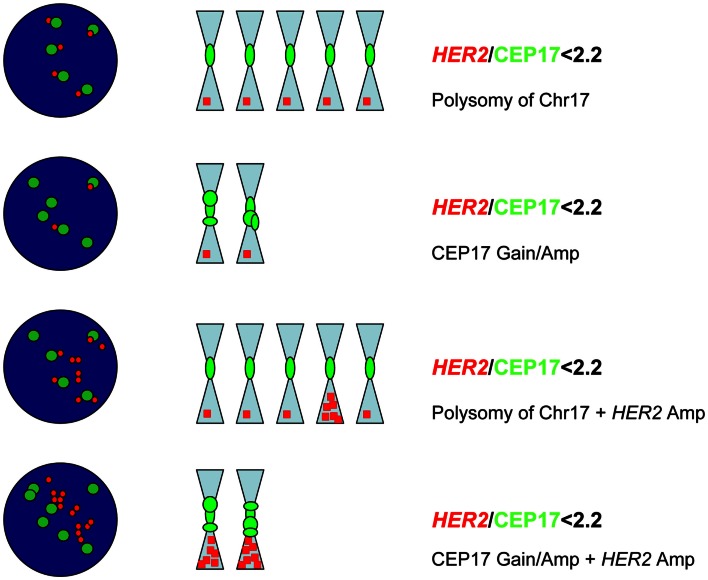
**Chromosome 17 polysomy *versus* CEP17 gain/amplification**. Possible scenarios in which additional CEP17 signals are encountered, from top to bottom: true chromosome 17 (chr17) polysomy without *HER2* gene amplification; gain/amplification of CEP17 without *HER2* amplification; *HER2* amplification in a context of chr17 polysomy; *HER2* amplification coupled with CEP17 gain/amplification. In all scenarios the ratio is below 2.2, however in the latter two cases *HER2* amplification is present (*HER2* mean copy number >6).

Assessment of *HER2* gene status by dual-color FISH can be troublesome sometimes (Isola et al., 2004; Troxell et al., 2006; Wolff et al., 2007; Marchio et al., 2009), in particular for tumors displaying abnormal copy numbers of CEP17 (Marchio et al., 2009). Polysomy of chromosome 17 defined by dual-color FISH as a mean of CEP17 copy number higher than three is observed in approximately 8% of all breast cancer specimens (Ma et al., 2005; Reddy et al., 2006; Wolff et al., 2007; Marchio et al., 2009), mostly among cases with four to six HER2 gene copies (the so-called “equivocal range” of FISH assessment) (Ma et al., 2005; Reddy et al., 2006; Marchio et al., 2009). However, we should keep in mind that polysomy is a cytogenetic definition and represents the occurrence in a nucleus of extra copies of one or more individual chromosomes (Marchio et al., 2009), therefore, *per se*, it can only be inferred from dual-color FISH on interphase nuclei (Figure 2). By coupling FISH and microarray-based comparative genomic hybridization (aCGH) analysis we have provided the first direct evidence that additional copies of CEP17 as detected by FISH are frequently caused by CEP17 gain/amplification (Figure 2) and that these phenomena are more prevalent than true chromosome 17 polysomy (38.9/55.5 *versus* 5.5%, respectively) (Marchio et al., 2009). This demonstration, subsequently validated by independent groups employing other techniques (Yeh et al., 2009; Moelans et al., 2010, 2011b; Varga et al., 2012), holds important clinico-therapeutic implications, as the occurrence of amplification of CEP17 can be responsible for misleading *HER2* FISH results due to the *HER2*/CEP17 ratio (Figure 2), precluding therefore anti-HER2 based therapy to some patients (Marchio et al., 2009).

One may ask whether, as pathologists, we are still doing a good job by following the ASCO/CAP recommendations and in particular the analysis of results based on the *HER2*/CEP17 ratio. Some (Troxell et al., 2006; Tse et al., 2011) have recommended the use of probes for additional chromosome 17 loci (*SMS* and *RARA* mapping to 17p11.2 and 17q21.2, respectively) as surrogate chromosome 17 controls in cases with a complex CEP17 FISH pattern, whilst others have claimed the use of aCGH in routine diagnostic practice (Yeh et al., 2009; Gunn et al., 2010). Although these techniques may provide additional information they do not provide a definitive answer in all of the cases (Marchio et al., 2009). We believe that results based upon *HER2*/CEP17 ratio are still a good indicator of *HER2* amplification, provided that in those cases harboring aberrant CEP17 copy numbers calculation is performed based on absolute *HER2* copy numbers (HER2 >6) (Viale, 2009).

In terms of misleading *HER2*/CEP17 ratio values a final remark should be made about another potential pitfall. It has been shown that *HER2* gene-amplified breast cancers with monosomy of chromosome 17 are poorly responsive to trastuzumab-based treatment (Risio et al., 2005). Indeed, a word of caution should be spelled out for those cases showing either chromosome 17 monosomy or loss of the short arm of chromosome 17 involving the centromeric region. In such a scenario the mean CEP17 copy number as calculated by FISH is consistently lower than 2, therefore a “diligent” application of the ratio, in presence of normal/low increase of *HER2* copy numbers, would lead to values higher than 2 or 2.2 without an underlying *HER2* amplification. As a consequence, in such cases *HER2* copy number should be taken into account instead of the ratio.

## HER2 Study in Diagnostic Practice in the Future: Possible Scenarios?

At present the long awaited update of ASCO/CAP guidelines are soon to be published, and we expect to face amendments in terms of scoring methods and cut-offs that may affect both pathology practice and oncology treatment decision making.

Beyond adjustments to scoring methods, breast diagnostic pathology may have to deal in the near future with two main issues, of which one is purely methodological and the other biological.

The methodological issue contemplates the possible introduction of new/alternative techniques to be incorporated in HER2 testing. Given the gray area of HER2 assessment as well as the recent description of CEP17 amplification that has generated skepticism about the *HER2*/CEP17 ratio, some have hypothesized that implementation of other assays would help sort out difficult cases.

Over the past 5 years or so, multiplex ligation-dependent probe amplification (MLPA) has emerged as a robust technique for HER2 testing. MLPA is a PCR-based technique that requires minute quantities of DNA isolated from FFPE material (50–200 ng) and uses multiple probes (up to 45) directed against target and control genes in each PCR run (Farshid et al., 2011; Moelans et al., 2011a). This technique was introduced in 2002 and over the years has gained widespread clinical acceptance for the identification of gene copy number changes in a broad range of genetic diseases to identify aneuploidy and trisomies, and more recently for *HER2* amplification (White et al., 2004; Jankowski et al., 2008; Kozlowski et al., 2008; Farshid et al., 2011). A probe set for the measurement of *HER2* gene amplification is commercially available (P004-C1 ERBB2 probemix MRC Holland). This probe set includes 49 MLPA probes of which 4 map to *HER2* (different segments of the gene), and 12 map to reference genes. MLPA has been compared with FISH/CISH and IHC with a concordance of 97% (Moerland et al., 2006) and 89% (Purnomosari et al., 2006) respectively, thus suggesting MLPA is a reliable technique to measure *HER2* amplification in breast cancer.

Potentially, MLPA offers some advantages over traditional techniques. First, the high throughput of a PCR-based assay has the appeal of scalability, which is important in terms of cost/effectiveness (Farshid et al., 2011). Second, access to another alternative testing assay for HER2 would be valuable for those cases (2–3%) that remain equivocal after the completion of both IHC and *in situ* hybridization testing and for discordant results between IHC and *in situ* hybridization techniques (Farshid et al., 2011), however specific studies focusing on this matter are yet to be carried out and MLPA has yet to be clinically validated (Moelans et al., 2011b). Moreover, some technical caveats should be mentioned, in particular: tissue morphology is lost and heterogeneity can be missed, finally tissue contamination may occur. Altogether these features should be carefully considered to avoid false-positive and false-negative results and as a best practice advice microdissection or mesodissection are recommended (Moelans et al., 2011b), best if performed based on IHC results.

In terms of biology of HER2+ tumors a “brand new” topic is the recent demonstration of another biological mechanism underpinning HER2 activation, i.e., activating mutations. Recent next generation sequencing studies have brought to the front-line the presence of *HER2* mutations in breast cancer, a phenomenon known since 2005 and that has been neglected due to the most pervasive mechanism of *HER2* gene amplification (Weigelt and Reis-Filho, 2013). Data from eight breast cancer genome-sequencing projects have identified 25 patients with *HER2* somatic mutations in cancers lacking *HER2* gene amplification (Kan et al., 2010; Banerji et al., 2012; Shah et al., 2012; Stephens et al., 2012; Bose et al., 2013). Most of the mutations affect the tyrosine kinase domain and some the ECD. Bose et al. (2013) have functionally characterized 13 HER2 mutations using *in vitro* kinase assays, protein structure analysis, cell culture, and xenograft experiments. Seven of these mutations were activating mutations and all of these mutations were sensitive to the irreversible HER2/EGFR tyrosine kinase inhibitor neratinib, thus validating *HER2* somatic mutations as drug targets for breast cancer treatment (Bose et al., 2013).

In the meantime we wait for these critical preclinical data to pave the way to HER2 sequencing-directed breast cancer clinical trials, the next challenge for pathologists will be to delineate the identikit of HER2 mutation carriers. From data reported by Bose et al. (2013) putative candidates seem to be identified with ER positive breast carcinomas (both ductal and lobular carcinomas analyzed), either HER2 negative (score 0/1+) or showing equivocal HER2 expression (score 2+).

We have therefore to come to terms that addiction to the continued activation of HER2 and its downstream signaling pathways may be determined by more than one mechanism (Weigelt and Reis-Filho, 2013) and pathologists may have soon to face the challenge to add a further step of investigation in this context.

## Conflict of Interest Statement

The authors declare that the research was conducted in the absence of any commercial or financial relationships that could be construed as a potential conflict of interest.
